# Light-Induced Pupillary Responses in Alzheimer's Disease

**DOI:** 10.3389/fneur.2019.00360

**Published:** 2019-04-12

**Authors:** Pratik S. Chougule, Raymond P. Najjar, Maxwell T. Finkelstein, Nagaendran Kandiah, Dan Milea

**Affiliations:** ^1^Department of Visual Neurosciences, Singapore Eye Research Institute, Singapore, Singapore; ^2^The Ophthalmology & Visual Sciences ACP, Duke-National University of Singapore (NUS) Medical School, Singapore, Singapore; ^3^Department of Neurology, National Neuroscience Institute, Singapore, Singapore; ^4^Duke-National University of Singapore (NUS), Singapore, Singapore; ^5^Singapore National Eye Centre, Singapore, Singapore

**Keywords:** Alzheimer's disease, dementia, pupillary light response, chromatic pupillometry, melanopsin expressing intrinsically photosensitive retinal ganglion cells, Parkinson's disease, post -llumination pupil response, cholinergic deficit

## Abstract

The impact of Alzheimer's disease (AD) on the pupillary light response (PLR) is controversial, being dependent on the stage of the disease and on the experimental pupillometric protocols. The main hypothesis driving pupillometry research in AD is based on the concept that the AD-related neurodegeneration affects both the parasympathetic and the sympathetic arms of the PLR (cholinergic and noradrenergic theory), combined with additional alterations of the afferent limb, involving the melanopsin expressing retinal ganglion cells (mRGCs), subserving the PLR. Only a few studies have evaluated the value of pupillometry as a potential biomarker in AD, providing various results compatible with parasympathetic dysfunction, displaying increased latency of pupillary constriction to light, decreased constriction amplitude, faster redilation after light offset, decreased maximum velocity of constriction (MCV) and maximum constriction acceleration (MCA) compared to controls. Decreased MCV and MCA appeared to be the most accurate of all PLR parameters allowing differentiation between AD and healthy controls while increased post-illumination pupillary response was the most consistent feature, however, these results could not be replicated by more recent studies, focusing on early and pre-clinical stages of the disease. Whether static or dynamic pupillometry yields useful biomarkers for AD screening or diagnosis remains unclear. In this review, we synopsize the current knowledge on pupillometric features in AD and other neurodegenerative diseases, and discuss potential roles of pupillometry in AD detection, diagnosis and monitoring, alone or in combination with additional biomarkers.

## Introduction

Dementia is a global epidemic and has become a public health priority. Alzheimer's disease (AD) is the most common cause for dementia worldwide (1), accounting for 50–70% of dementia cases. The two major neuropathological landmarks of AD are deposition of insoluble amyloid-β (Aβ) plaques and formation of neurofibrillary tangles, composed of hyperphosphorylated *tau* proteins. These pathologic abnormalities are found in the central nervous system, as well as in the retina (2–4). The pathophysiology of AD is poorly understood, but a common hypothesis postulates that aggregation of Aβ is a pre-requisite for tau accumulation, neurodegeneration, and ultimately, to clinical manifestations. Clinical features of AD include progressive cognitive decline, affecting memory, learning, language, visuospatial abilities, and executive functions, but also deterioration of sleep and normal circadian rhythms (5). Most often extensive and largely irreversible neuronal histopathological changes precede clinical features of AD (6), which may explain the current failure of all disease modifying agents in this condition. For these reasons, it is believed that early diagnosis of AD is crucial for early and effective therapeutic interventions, improving AD outcomes.

Several *in vivo* biomarkers have been proposed for early identification of AD pathology, including brain imaging biomarkers (positron emission tomography after Aβ labeling) (7), as well as fluid biomarkers (within the cerebrospinal fluid and, possibly, in the blood) (8). These, and other novel genetic, biological, deep-learning based, or behavioral biomarkers aim to surpass the current performance of classical clinical evaluations in AD, which are subjective, time-consuming and deliver variable results. The eye, which is embryologically, anatomically, and physiologically an extension of the brain, has been an early explored target for identification of neurodegeneration biomarkers in AD (9, 10). Functionally, various ocular biomarkers have been tested for detection and evaluation of AD, such as eye movement recordings and pupillary responses to light or to cognitive load (9–11). Several studies have suggested that AD may be associated with altered pupillary light responses (PLR), as a consequence of abnormalities in the retina and/or the efferent pupillary system. Several arguments support the possible pupillary involvement in AD, including pathological changes in the retina, as well as existence of parasympathetic (cholinergic) and sympathetic (adrenergic) dysfunctions in the disease. Indeed, neurodegeneration commonly affects the locus coeruleus (LC), located in pons and involved in the sympathetic control of pupil size and PLR (2, 12), as well as the Edinger Westphal nucleus (EWN) (4, 13), involved in the parasympathetic control of the pupil. Pupillometry is an easy, non-invasive and affordable tool, allowing the evaluation of the PLR in AD and other ocular and neurological diseases. Whether static or dynamic pupillometry yields useful biomarkers for AD screening or diagnosis remains unclear. In this review, we synopsize the current knowledge on pupillometric features in AD and other neurodegenerative diseases, and discuss potential roles of pupillometry in AD detection, diagnosis and monitoring, alone or in combination with additional biomarkers.

## The Neurophysiology of the Pupillary Light Response

### Afferent and Efferent Pathways Governing the Pupillary Light Response

**Figure 1:**
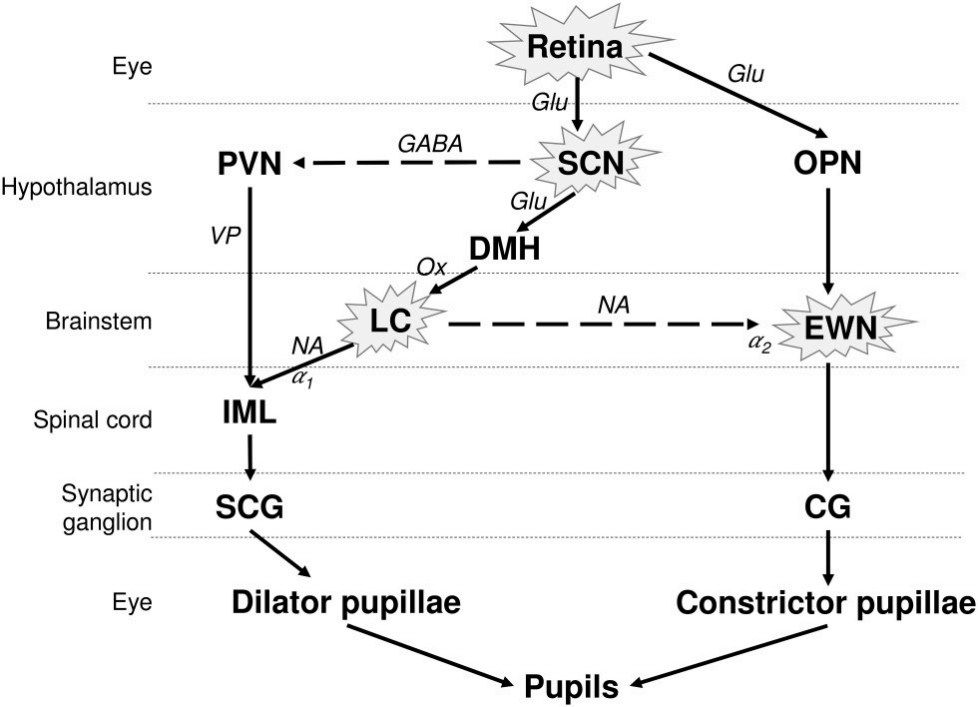
Neuronal control of the PLR and affected structures in AD. The pupil size depends on the interplay between antagonistic parasympathetic and sympathetic impulses. The photoreceptors in retina, including the melanopsin expressing retinal ganglion cells (mRGCs), stimulate the OPN, connected to the parasympathetic EWN. mRGCs also project to the SCN, which is connected to the sympathetic LC. EWN is the major cholinergic center subserving the pupillary constriction to light. Light has an inhibitory effect on sympathetic activity and PVN via SCN. This inhibitory effect is attenuated by sympatho-excitation, which is mediated via the SCN and LC (14). The encircled structures are affected in AD. Solid lines, excitatory connections; dashed lines, inhibitory connections; Hypothalamic nuclei: SCN, suprachiasmatic nucleus; PVN, paraventricular nucleus; DMH, dorsomedial hypothalamus. Autonomic premotor nuclei: OPN, olivary pretectal nucleus; LC, locus coeruleus. Parasympathetic nucleus/ganglion: EWN, Edinger Westphal nucleus; CG, Cilliary ganglion. Sympathetic nucleus/ganglion: IML, intermedio-lateral column of spinal cord; SCG, superior cervical ganglia. Neurotransmitters: Glu, glutamate; GABA, γ-amino-butyric acid; VP, vasopressin; Ox, orexin; Ach, acetylcholine; NA, noradrenaline. Adrenoceptors: α1, excitatory; α2, inhibitory.

The pupil size is under the control of a closed autonomic loop. The pupil constrictor and dilator muscles receive antagonistic impulses from the parasympathetic (cholinergic) and sympathetic (adrenergic) autonomic nervous systems, respectively (Figure 1). The PLR is also dependent on the integrity of the retina, and in particular on the integrity of the intrinsically photosensitive melanopsin expressing retinal ganglion cells (mRGCs) (15). Although the mRGCs are activated by rods and cones, they are also intrinsically photosensitive through the melanopsin photopigment, subserving the PLR via central projections to the olivary pretectal nucleus (OPN) (16), which projects to the EWN, as demonstrated in non-human primates. The parasympathetic EWN is a cholinergic nucleus in the oculomotor complex, at the origin of preganglionic efferent neurons which synapse in the ciliary ganglion, subsequently innervating the constrictor pupillae (14, 17). The mRGCs also project to the central circadian clock located in the hypothalamic suprachiasmatic nucleus (SCN), which governs various bodily circadian rhythms and projects to the sympathetic locus coeruleus (LC), located in pons (Figure 1) (18, 19) [for review see (20)]. The sympathetic efferent system, including LC and SCN, regulates the resting pupil size at different levels of background illumination (17), by controlling the tone of the dilator muscle. Beyond the intervention of these motor structures, the pupillary size and function can be modulated by supranuclear neuronal influences. Other possible confounding factors, affecting the size of the pupils include the respiration rate (21), emotional status (22), vigilance (23), and age (24).

### Retinal Photoreception and Chromatic Pupillometry

Recorded using an infra-red pupilometer, the PLR is governed by rods, cones and mRGCs. The intrinsically photosensitive mRGCs, located in the inner retina, produce a sustained constriction of the pupil in response to bright blue light which persists even after light offset; in addition to integrating signals from rods and cones (25). Different light wavelengths, at different intensities stimulate specifically different retinal photoreceptors (26–29). Thus, chromatic pupillometry, using different wavelengths and light intensities has been used for the evaluation of inner and outer retina integrity, in various conditions (26–29).

## Pathophysiology of Light-Induced Pupillary Responses in AD

### Cholinergic Deficit in AD

Loss of cholinergic neurons is a common event in AD, possibly leading to alteration of cognitive processes. Specific loss of cholinergic neurons, mainly located in the medial septum and in the para-hippocampal area, is associated with memory impairment (30), but also with other cognitive deficits seen in AD (31, 32). The hypothesis of cholinergic deficit fails however, to explain other impairments in AD, i.e., disruption of circadian rhythms, sleep, and executive functions.

AD affects the cholinergic EWN, which is the central brainstem sub-nucleus of the oculomotor complex, involved in the control of the pupil constriction. Pathologic studies have shown that the EWN is affected even at early stages of AD, displaying deposition of Aβ amyloid plaques and neurofibrillary tangles (4, 13, 33). AD is associated with increased glutaminyl cyclase activity, resulting in formation of highly neurotoxic Aβ amyloid precursors (pyroglutamate Aβ peptide), identified in the EWN and in the preganglionic cholinergic PLR-governing neurons, as well as in other cholinergic neurons in the nucleus basalis of Meynert (34). In AD, the EWN neurons, display a decrease in their total dendritic length per neuron as well as a severe loss of distal dendritic branches and dendritic spines, leading to severe decrease in synaptic contacts (35). It has been suggested that these pathological changes of the EWN may be an early and specific feature of AD and they may result in decreased cholinergic control of pupillary responses (35). Unfortunately, little is known about the involvement of other parasympathetic structures involved in the PLR, i.e., the olivary pretectal nucleus and ciliary ganglia.

### Adrenergic Deficit in AD

The LC modulates pupil size in 2 possible ways; by direct stimulation of preganglionic sympathetic neurons, as well as by inhibitory regulation of the EWN (Figure 1) (17, 36). Various factors stimulating the LC may modulate the PLR. For example, anxiety, associated with excitation of the LC (37, 38) or drugs increasing noradrenergic output to the EWN like noradrenaline re-uptake inhibitors (14, 39), lead to an increased sympathetic response on PLR like prolonged latency, reduced amplitude of constriction and faster redilation. Conversely, drugs inhibiting LC activity like clonidine (α2-adrenoceptor agonist) cause pupillary miosis and reduce the sympathetic effect on PLR (40–42). In monkeys, an electrophysiologically detectable activity in LC has been associated with mydriasis at rest (43).

Patients with AD and mild cognitive impairment (MCI), considered as the pre-clinical stage for AD, undergo significant loss of noradrenergic neurons in the LC (55 and 30%, respectively), compared to healthy controls, a finding which may impact the PLR (44). Neuronal loss in the LC of patients with AD may lead to decreased sympathetic supply to the iris and reduce the baseline pupil size (45).

### Retinal Changes in AD

Aging is associated with optical (46, 47) photoreceptoral and retinal neuronal changes (48–50). Optically, in spite of decreased lens transmittance for short wavelength blue light in aging and cataract, the mRGCs induced pupillary response by blue light are well-preserved (51–53), and the pupillary responses are reduced irrespective of the wavelength of light. Although, aging has been associated with axonal and retinal ganglion cell loss (54), AD has been associated with greater thinning of retinal nerve fiber layer compared to age matched healthy controls (55–57), suggesting an accelerated loss of RGCs in AD patients. Pathological studies have shown presence of Aβ amyloid plaques in the retina of AD patients (58, 59) including in the inner layers of retina (3). These changes were often associated with blood vessel abnormalities and areas of cellular degeneration, similar to what is seen histologically in the brain of patients with AD (60, 61).

Moreover, retinas of patients with advanced AD display not only histological evidence of mRGC loss, but also selective deposition of Aβ amyloid plaques within these cells, which subserve the PLR (3). Less is known about the early selective loss of mRGC and possible mRGC Aβ deposition, occurring in AD. Occurrence of such a phenomenon should allow discrimination between normal aging patients and AD, using chromatic pupillometry. Chromatic pupillometry has been used in other conditions as a marker of mRGC integrity (62). In primary open angle glaucoma, a condition associated with histological mRGC loss (63), abnormal melatonin secretion profile (64) and sleep and circadian rhythm dysfunction (65), various pupillometric studies have shown abnormal PLR responses (28, 66–70). Conversely, in mitochondrial hereditary optic neuropathies, mRGCs are resistant to neurodegeneration, explaining the relatively preserved chromatic pupillometry parameters (71–73) and melatonin profiles (74). It is possible that mRGC loss, alone or combined with neuronal loss occurring in the suprachiasmatic nuclei, may be associated with circadian rhythm dysfunctions which can occur even at early stages of AD (3, 5).

## Features of the Pupillary Light Responses in AD

### Baseline Pupil Diameter

The consequence of AD on the pupillary diameter at rest has been controversial in various studies, probably due to methodological differences, i.e., measurement conditions and sample sizes. A few studies with small sample size (range 9 to 23 AD patients) have reported reduced baseline pupillary diameters in AD compared to those of healthy subjects (45, 75–77). Other studies did not find any difference in baseline pupil diameters between AD, MCI and controls (78, 79), however, the groups were not age-matched in the study with largest sample size (*n* = 66 AD, 42 MCI) (78), while AD patients in the other study were using cholinergic and anti-depressant medications which may alter the baseline pupil size (*n* = 15 AD) (79). An increased baseline pupillary diameter has also been reported in AD (*n* = 20 AD), but no details of age of the two groups were mentioned (80) (Table 1).

**Table 1:** Summary of studies on PLR in AD.

Study (*n* = sample size)^*^	**Light paradigm**	**BPD**	**LoC**	**AC**	**MCV**	**MCA**	**Redilation velocity**	**Comments and features of parasympathetic (PSD) and sympathetic (SD) deficiencies**
Prettyman et al. (45), (*n* = 9)	11 × 200 ms 565 nm flashlights at 8.5 × 10^−3^ and 7 × 10^−2^ mW/cm^−2^, 0.43 and 1.84 mW/cm^−2^ at 1 cm from the eye	↓	↔	↓	NA	NA	↑	PSD features: ↓ AC and ↑redilation SD features: In darkness, pupillary dilation amplitude and velocity decreased, along with decreased BPD.
Ferrario et al. (80), (*n* = 20)	1 s of 660 nm flashlight	↑	↑	↔	↔	↑	↔	PSD: ↑BPD and ↑LoC SD: ↑MCA Limitations: Age of different groups not mentioned.
Fotiou et al. (81), (*n* = 5)	20 ms flashlight delivered using a xenon lamp at 30 cm from the eye	↑	↔	NA	NA	NA	NA	PSD: ↑BPD. Cholinergic medications reduced BPD close to controls.
Granholm et al. (79), (*n* = 15)	16 × 150 ms pulses of light at 20 and 40 lux from a computer screen at 77 cm	↔	NA	↓	NA	NA	NA	PLR checked after diluted tropicamide test. 9 AD patients were using cholinergic medications and 5 were using anti-depressant medications.
Fotiou et al. (75, 76), (*n* = 23)	20 ms flashlight delivered using a xenon lamp at 30 cm from the eye	↓	↑	↓	↓	↓	↑	PSD: ↓AC, ↓MCV, ↓MCA, ↑LoC, ↑redilation SD: ↓BPD
Frost et al. (77), (*n* = 19)	31 ms white flash at 180 μW	↓	↔	↓	↓	↓	↑	PSD: ↓ Mean constriction velocity, ↓MCV, ↓MCA, ↓AC and ↑% redilation at 3.5 s SD: ↓BPD
Bittner et al. (78), (*n* = 66AD, 42MCI)	40 × 200 ms pulse of 585 nm light at 200 cd/m^2^	↔	↔	↔	NA	NA	NA	Controls were younger than AD and MCI patients and had greater constriction amplitude on repetitive stimulations
Fotiou et al. (82), (*n* = 42)	20 ms flashlight at 24.6 cd/m^2^	NA	↑	NA	↓	↓	NA	PSD: ↓ MCV, ↓MCA, ↑LC MCV and MCA correlated well with MMSE scores.
Frost et al. (83), (*n* = 14)	31 ms white flashlight at 180 μW	NA	NA	↓	↓	↓	NA	Limitations: Controls were younger than AD patients.
Van Stavern et al. (84), (*n* = 24)	3 × 525 ms white flashlight at 180 W	NA	↔	↔	NA	NA	↔	Preclinical AD patients with no cognitive impairment were studied.

BPD, baseline pupillary diameter in mm; LoC, latency of constriction in seconds; AC, amplitude of constriction; MCV, Maximum constriction velocity; MCA, maximum constriction acceleration; AD, Alzheimer's disease; PSD, Parasympathetic deficiency; SD, Sympathetic deficiency; ↓, decreased; ↑, increased; ↔, Not significant; NA, Not applicable/available; PLR, pupillary light response; MMSE, Mini-Mental State Examination;

* *n, Sample size of patients included in the study (excluding controls)*.

### Constriction Phase

**Figure 2:**
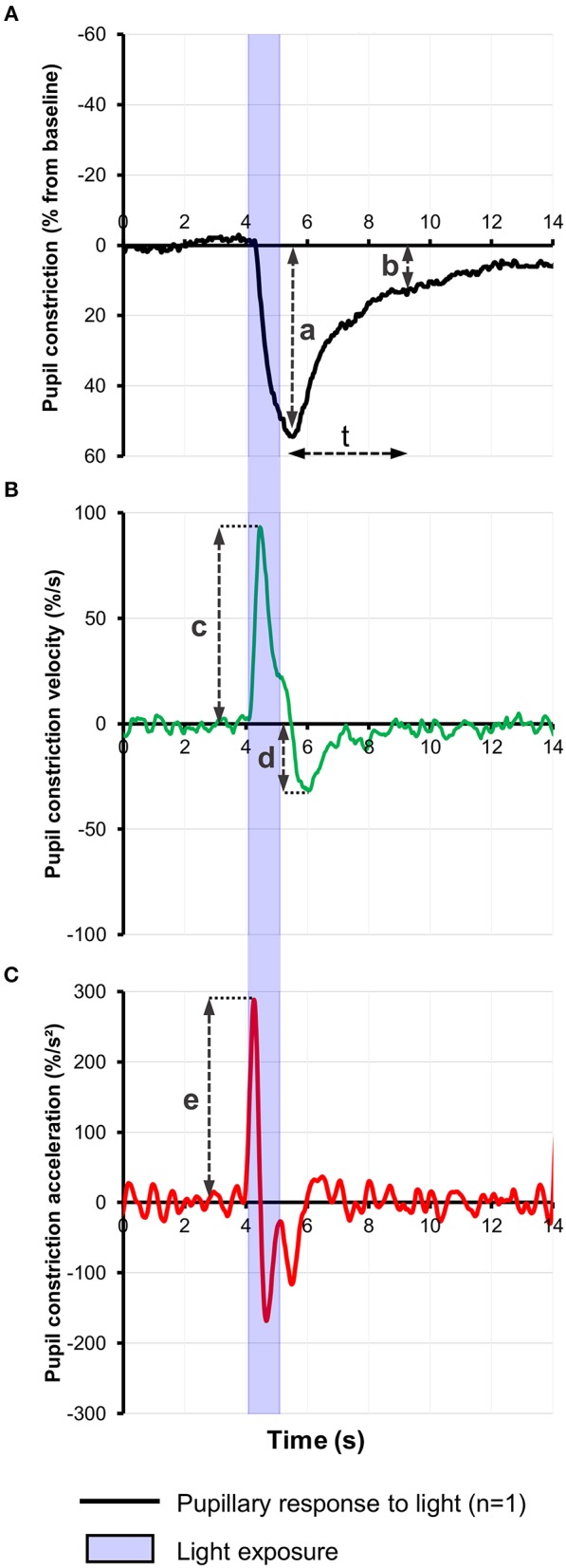
**(A)** Representative baseline-adjusted pupillary response to a 1-s bright blue light (480 nm) stimulus of 13.0 Log photons/cm^2^/s in a healthy individual. Pupil constriction velocity **(B)** and acceleration **(C)** curves were computed, respectively, from the trace in **(A)** as the first and second derivatives of change in pupil constriction with respect to time. Pupillometric features extracted include: a, amplitude of pupillary constriction; b, post-illumination pupillary response after (t) seconds from light offset; c, Maximum constriction velocity (MCV); d, maximum redilation velocity; e, Maximum constriction acceleration (MCA).

Most pupillometric studies in patients with AD have reported results compatible with parasympathetic deficiency, translating to increased latency of pupillary constriction to light, decreased constriction amplitude, reduced mean constriction velocity and faster redilation after light offset (45, 75, 76, 83, 85) (Table 1). Pupil constriction velocity is obtained as the first derivative of change in pupil size with respect to time and acceleration as the second derivative (change in constriction velocity with respect to time) (Figure 2). Patients with AD typically display decreased maximum velocity of constriction (MCV) and maximum constriction acceleration (MCA) compared to controls, suggesting a parasympathetic deficiency. Amongst all pupillometric features, MCA and MCV have been reported as the most accurate parameters to differentiate AD patients from healthy controls (75, 76, 83). Nonetheless, other studies have failed to find such differences between AD patients and healthy individuals (78, 80). These differences may be the result of the different illumination protocols used, since studies using white light typically are associated with larger constriction amplitudes and shorter latencies (24), compared to studies using red light at 660 nm (80) or 585 nm (78). Considering that different studies have used different intensity and wavelength stimuli, this effect cannot be completely attributed to the wavelength alone. However, other studies have demonstrated that when photon density is kept constant, shorter wavelength light produced greater constriction amplitude than longer wavelength (86, 87).

In a recent study in cognitively normal pre-clinical AD subjects, diagnosed on the basis of high cortical binding potential of Aβ amyloid on PET imaging and/or low CSF Aβ amyloid levels, there was no significant difference in any of the pupillary parameters in response to light emitting diodes (LEDs) producing a white flash of 180 W for 525 ms, compared to healthy controls (84). Only a marginal difference was observed in PLR in pre-clinical stages, suggesting that the effect of AD on PLR in pre-clinical stages is very small and may not be detectable. This suggests that PLR using achromatic light stimulus may be a valid biomarker for established Alzheimer's disease but it may have limited clinical utility in screening for AD in pre-clinical stages, perhaps due to the very small disease effect on PLR (84).

A pupillometric study, using non-Maxwellian retinal stimulation with repetitive, brief, long wavelength light (585 nm), aimed to compare patients with MCI, AD and normal controls, but failed to find inter-group differences (evaluating baseline diameters, constriction amplitudes, and constriction latencies), after adjustment for age (78). However, the use of specific repetitive light stimuli induced stronger pupillary responses in controls in terms of relative constriction amplitude (difference in the PLR measurements of the first and last stimuli), compared to AD and MCI patients, suggesting that repetitive pupillary stimulation might be a more appropriate stimulus to discriminate patients with AD and MCI from controls (78). It was proposed that repetitive stimuli caused a fatigue of the sympathetic inhibitory system to the parasympathetic pathway revealing the true effect of parasympathetic system alone on PLR. Smaller relative miosis and amplitude of constriction in AD patients suggests a decreased parasympathetic innervation to the pupillary system which may not be detected in the presence of inhibitory sympathetic system. Although relative amplitude was independent of age, the difference between MCI and control groups was not statistically significant while between AD and controls may fail to reach statistical significance if Bonferroni correction is applied. Moreover, its definition and calculation is not clearly explained in the study. Constriction phase due to different wavelength light stimuli in AD patients has not been studied yet.

### Pupillary Redilation Phase

The pupillary redilation phase has been explored in several studies, but these results are sometimes difficult to compare, given the variable definitions of this parameter such as (i) percentage of pupillary redilation after 3.5 s of white flash light offset (75–77), (ii) 75% redilation time (45, 77), and (iii) average dilation velocity (77, 84). The majority of these studies have reported a faster pupillary redilation phase [analogous to decreased Post-illumination pupil response (PIPR)] in AD patients compared to controls, making it the most consistent PLR feature in AD patients (45, 75–77). In a study which extensively studied the redilation phase, the percentage recovery at 3.5 s and the 75% redilation time was significantly greater in AD patients compared to aging controls. Although the mean dilation velocity (mm/sec) was slower in AD patients, it can be ignored since the pupil size was not calculated as a percentage of baseline pupil diameter which was significantly smaller in AD (77). A recent study in preclinical AD alone did not find a significant increase in dilation velocity compared to controls following an achromatic light stimulus, however, it is not clear whether the measurements were adjusted to baseline pupil diameter (84). The faster pupillary redilation after light offset has been attributed to the diminished parasympathetic tonus associated with the cholinergic deficit, translating into failure to maintain a tonic pupillary constriction. An alternative explanation, which was not yet considered in previous studies, is that the accelerated pupillary redilation may be the result of selective mRGC loss in AD, causing an abnormal, faster PIPR as seen in other conditions affecting the mRGC, such as glaucoma (66). This hypothesis is in line with the findings of another study, which did not find any difference of the redilation phase between a group of AD patients and controls, after exposure to red light (660 nm) (80). Indeed, red light at medium high intensities is less prone to stimulate the mRGC and might have failed to explore their dysfunction. Although, redilation was not found to be different in preclinical AD patients in a recent study using a 180 W achromatic stimulus for 525 ms (84), chromatic pupillometry studying different parameters like constriction amplitude or PIPR to blue light, can still be a viable option in such cases due to selective mRGCs loss as reported in AD patients (3). No study till date has investigated pupillometric signs of mRGC dysfunction or impaired PIPR to blue light in AD patients and needs to be explored in the future. However, these interpretations are speculative, it is well-possible that the faster redilation in AD patients may be the result of an interplay between the two factors, i.e., the mRGC loss and the cholinergic deficit. It is noticeable that mRGC loss in AD should result in selective faster redilation after offset of blue light (460 nm), which specifically stimulates the mRGCs. On the opposite, faster redilation due to cholinergic deficiency should be independent of wavelength of stimulus used, occurring after offset of both blue and red light. Future studies taking into account these factors, should be able to disentangle the respective role of the afferent vs. efferent system in the pupillary redilation phase.

## A Potential Role for Pupillometry in a Multi-Modal Approach for Detecting AD?

### Generic Screening and Diagnostic Tests for AD

The gold standard for AD diagnosis is still based on pathology findings. However, in clinical practice, screening for AD or cognitive impairment uses various questionnaires and interactive tests, including the Mini Mental Scoring Examination (MMSE) or the Montreal cognitive assessment test (MoCA). These tests have numerous limitations, including language barriers, geographical adaptability, subjectivity, long implementation time, and the necessity for constant supervision by trained and skilled personnel. Several objective tests have been developed for early AD detection and diagnosis including cerebro-spinal fluid (CSF) analysis to measure beta amyloid (Aβ), total tau proteins and phosphorylated tau peptides quantification (8, 88), and magnetic resonance imaging (MRI) imaging of the brain and positron emission tomography (PET) scan measuring the brain Aβ plaque burden (6, 7, 89). These modalities are expensive, invasive and potentially dangerous (90, 91). In addition, they detect only structural and not functional changes in AD.

From the published studies, pupillometric evaluations may not suffice for early AD detection. However, they may constitute an adjunctive method in a, multimodal approach, combining (i) novel, cognitive, visuospatial, and memory tests involving portable virtual reality devices, (ii) retinal imaging for detection of neuronal loss and/or amyloid deposition, and (iii) objective functional outcomes provided by targeted color pupillometry.

### PLR and Genetic Mutations

Apolipoprotein E is a fat-binding protein involved in the metabolism of fat, produced by APOE gene found on chromosome 19, being the only genetic factor associated with the common late onset AD. APOE mutation is not a causative mutation, but is rather considered as a risk factor for AD (92, 93). Although the PLR is not directly influenced by the APOE ε4 carrier status (78), their combination may increase the area under the curve for the combined test performance (83).

A pupillometric study has evaluated participants from a single family harboring an Amyloid-Beta Precursor Protein genetic mutation (APPGlu693Gln) (6 carriers with no cognitive impairment and 6 non-carriers) (94). This mutation results in a rare form of autosomal dominant Alzheimer's disease with phenotypical penetration approaching 100% and which is responsible for an early onset of AD. The pupillometric assessment yielded a slower pupil 75% recovery time in mutation carriers compared to non-mutation carriers. Globally, pupillometric changes were detected in pre-symptomatic carriers of the mutations, but were not statistically significant.

### PLR and Cognitive Assessment Tools

MMSE is routinely used to screen elderly subjects for dementia and has a AUROC of 0.89 (95). PLR in patients with AD having higher MMSE and Wechsler Memory Scale (better cognition) scores correlated moderately with MCV, MCA and latency of constriction (82). On repetitive pupillary stimulation, higher MMSE correlated with larger increase in amplitude and relative amplitude and greater decrease in the latency (p < 0.05) of constriction (78). These outcomes suggest that the pupillary light response may depend on the severity of the disease and can be used for monitoring the disease progression. However, combined efficacy of MMSE and PLR has not been explored as both tools are practical, easy, non-invasive, and affordable and may yield better accuracy if combined together, compared to individual outcomes.

### PLR and CSF Abnormalities

Decrease in pupillary constriction amplitude with repetitive stimulation in AD patients correlated with lower Aβ42 protein levels (*p* = 0.01) and a trend with higher tau levels in CSF (*p* = 0.08) (78). This suggests a possible association between cholinergic deficit, decreased Aβ42 protein levels and a trend with higher tau levels in CSF which supports a causative role of Aβ amyloid plaques in central cholinergic deficit (92). To date, the efficacy of a combined PLR-CSF screening method remains unknown.

### PLR and Topical Weak Anticholinergic Eye Drops

In a highly controversial study, Scinto et al. reported that patients with AD exhibit larger pupil dilation compared to age-matched controls after instillation of diluted anticholinergic eyedrops (Tropicamide 0.01%) (96) Several studies have contradicted this finding (97–99) which could be due to ethnicity, age, ocular penetration of drug, properties of the solution and background luminance (79). A combination of topical weak anticholinesterase and PLR showed significant reduction in constriction amplitude for AD and Parkinson's patients compared to controls, but no significant difference between Alzheimer's and Parkinson's patients was found, while latency of constriction was similar within the 3 groups (79). However, others did not find any such significant difference in PLR pre or post weak anticholinergic eyedrops use, between AD patients and controls (80). Hence, the use of weak anticholinergic eyedrops may not improve the efficacy of PLR in detecting AD, since it may not give consistent results and decrease in amplitude of constriction is noted in AD even without using topical anticholinesterase.

## Limitations of Previous Pupillometric Studies in AD

Most of the previously published pupillometric studies in AD have various methodologic limitations. The intensity, light wavelength and duration of light exposure were variable in all the above mentioned studies. Yet, these parameters can affect, independently, or in combination, the PLR outcomes (24, 100). Therefore, there is a high need for standardization of experimental conditions in AD studies, similar to what has been described in studies using light therapy (101) and in other animal studies (102). Most current pupillometric studies agree of the need for standardized analysis of baseline pupil diameters (103, 104). Interestingly, most of the previous PLR studies in AD have not normalized the baseline diameter in their subjects, making any comparison very difficult. In a few studies evaluating pupillometric results in AD, there was no age-matching between the groups of patients and controls (78, 79, 83). Indeed, the decreased pupillary diameter with age (105, 106) may constitute a confounding factor. Last, but not least, the severity of AD was rarely taken into account in the evaluation of the PLR.

### Effect of Cholinergic Medications

Only a few, small sample studies have reported the effect on the PLR of cholinergic drugs, commonly used in AD (79, 81). Thus, AD patients without cholinergic medications displayed larger baseline pupillary diameter, reduced pupillary miosis and higher number of oscillations at rest, compared to AD patients on cholinergic treatments and to healthy controls. Patients on medications had a greater latency of onset of constriction compared to both the controls and the medication free AD patients (81). However, other study in AD patients, have not found an effect of cholinergic medications on pupillary miosis but supported the increase in latency of constriction (79). PLR in AD patients on cholinergic medications behaved more like controls with no significant difference in constriction amplitude and baseline pupil diameter than their medication free counterparts (81). Taken together, these findings suggest that cholinergic medications might improve the pupillary responses in AD patients. Due to the very small sample size of these studies, it is difficult to conclude regarding a possible effect of cholinergic medications on the PLR in these patients. Additional studies are needed to understand the effect of cholinergic medications on PLR.

### Effect of Ocular Co-morbidities

The most common ocular condition associated with aging is cataract which can attenuate the PLR response to both red and blue light. But the senescence of the lens does not selectively reduce the mRGCs responses to intense blue light and is well-preserved, in spite of its decreased lens transmittance in aging and cataract (51). Different retinal and optic nerve conditions can affect PLR and using chromatic pupillometry it is possible to localize the loss of photoreceptoral function i.e., inner or outer retina (107). Primary open angle glaucoma is associated with decreased PLR in response to exposure to both red and blue light with decreased PIPR for blue light (28, 70), while retinal dystrophies affecting rods and cones lead to decreased PLR responses to red and low intensity blue light with an increased PIPR to bright blue light stimulus (108, 109). Diabetic retinopathy and age-related macular degeneration can also affect the PLR (110, 111), but there is little indication to what extent these PLR alterations might be disease-specific, or whether they may confound co-existence of AD in the aging population.

## PLR in Other Neurodegenerative Disorders

Autonomic nervous system dysfunction has been described in Parkinson's disease (PD) (112), including cholinergic deficit (113), Various PLR abnormalities have been described in PD, including reduced amplitude of constriction, increased latency and decreased velocity and acceleration of constriction, while the baseline pupil diameter may be increased or not significantly different compared to healthy controls (Table 2) (76, 114). Pupillary unrest has also been increased in PD patients which were not on medications compared to healthy individuals or in treated patients (115), Pupillary redilation has not been significantly different in PD studies using white flash light stimuli (114, 115). However, a recent chromatic pupillometry study in PD patients has suggested that the PIPR following a short wavelength blue light elicits a faster redilation compared to healthy controls. This finding is consistent with loss of mRGCs in PD (116), possibly related to deposition of α-synuclein in the retinal ganglion cells in the inner plexiform layer of the retina (117, 118). An alternative explanation might be related to reduction in the dopamine expression in the amacrine cells which relay information from rods and cones to mRGCS.

**Table 2:** Summary of studies on PLR in PD.

Study (*n* = sample size)^*^	**Light paradigm**		**BPD**	**LoC**	**AC**	**MCV**	**MCA**	**Redilation velocity**	**Comments and features of parasympathetic (PSD) and sympathetic (SD) deficiencies**
Micieli et al. (114), (*n* = 23)	500 ms flashes of white light at 1,400 lux		↔	↑	↓	NA	NA	↔	BPD in dark was not significantly different from controls but in photopic conditions, pupillary diameter was significantly larger in PD patients. PSD: ↑LoC, ↓AC
Granholm et al. (79), (*n* = 15)	16 × 150 ms pulses of light at 20 and 40 lux from a computer screen at 77 cm		↔	NA	↓	NA	NA	NA	PLR checked before and after diluted tropicamide test.
Fotiou et al. (76), (*n* = 22)	20 ms flashlight delivered using a xenon lamp at 30 cm from the eye		↔	↑	↓	↓	↓	NA	PSD: ↓AC, ↓MCV, ↓MCA, ↑LoC, ↑redilation SD: ↓BPD
Jain et al. (115), (*n* = 17)	11 × 1 s white flashlight at 13 cd/m^2^, subtending a visual angle of 4.60° at a distance of 73 cm		NA	NA	NA	↔	NA	↔	Pupillary unrest was significantly higher suggestive of autonomic dysfunction. Five patients were on dopaminergic medications.
Joyce et al. (116), (*n* = 17)	8 s pulsed and 12 s 0.5 Hz sinusoidal stimulations using 465 nm and 638 nm lights at 15.1 log photons/cm^2^.s at the corneal level	Blue Red	NA NA	NA NA	↔↓	NA NA	NA NA	↑↔	Selective faster redilation to short wavelength light suggests mRGC dysfunction.

BPD, baseline pupillary diameter in mm; LoC, latency of constriction in seconds; AC, amplitude of constriction; MCV, Maximum constriction velocity; MCA, maximum constriction acceleration; PD, Parkinson's disease; PSD, Parasympathetic deficiency; SD, Sympathetic deficiency; ↓, decreased; ↑, increased; ↔, Not significant; NA, Not applicable/available; mRGC, Melanopsin expressing retinal ganglion cells; PLR, pupillary light response;

* *n, Sample size of patients included in the study (excluding controls)*.

Autonomic nervous system dysfunction has also been described in dementia with Lewy bodies and to a lesser extent in fronto-temporal dementia, which can be associated with retinal abnormalities (119, 120). However, the specific effects of autonomic dysfunctions and retinal changes on pupillary light reflexes have not yet been studied in these disorders.

## Summary

In summary, MCV and MCA appear to be the most accurate PLR features, but also the least studied, while redilation velocity/rate (corresponding to PIPR) appears to be the most consistently altered PLR feature in AD. In conjunction with other features (baseline pupillary diameter, amplitude and latency of constriction), these parameters predominantly suggest parasympathetic deficiency, associated with mRGCs dysfunction. Longitudinal and adequately designed studies are necessary to validate the use of pupillometry in the early detection and follow-up of AD. Further studies are needed to establish the respective contribution of retinal (afferent) vs. efferent pupillary pathways in the alteration of the pupillary responses, for which chromatic pupillometry can potentially be used and translated into clinical application. Studies may also be designed to investigate the effect of cholinergic medication on PLR in AD patients and the potential use of artificial intelligence on pupillometric traces and video recordings. Using low-cost hardware, pupillometry can now easily be implemented in both remote tele-ophthalmology settings (121), as well as in continuous home monitoring (122). Combined with cognitive game-based investigations and wearables (123, 124), pupillometry may allow a more accurate screening, follow-up, and management of patients with AD.

## Author Contributions

PC conducted the review of literature. PC, DM, and RN wrote the manuscript. All authors (PC, RN, DM, MF, and NK) reviewed and approved the manuscript.

### Conflict of Interest Statement

DM has a patent application based on a pupillometry protocol (PCT/SG2015/050494): A method and system for monitoring and/or assessing pupillary responses. DM and RN have a patent application based on a hand held device for ophthalmic and neurological screening (PCT/SG2018/050204): Hand held ophthalmic and neurological screening device. The remaining authors declare that the research was conducted in the absence of any commercial or financial relationships that could be construed as a potential conflict of interest.
